# Virucidal effect of povidone iodine on COVID-19 in the nasopharynx: A structured summary of a study protocol for an open-label randomized clinical trial

**DOI:** 10.1186/s13063-020-04963-2

**Published:** 2021-01-04

**Authors:** Mohammad Jahid Hasan, S. K. Nurul Fattah Rumi, Sultana Sahana Banu, A. K. M. Nasir Uddin, Md Shahnoor Islam, Mostafa Kamal Arefin

**Affiliations:** 1 Pi Research Consultancy Center, Dhaka, Bangladesh; 2 Dhaka Medical College, Dhaka, Bangladesh

**Keywords:** COVID-19, Randomized clinical trial, Protocol, Povidone Iodine, Nasal spray, Nasal Irrigation, DMC

## Abstract

**Objective:**

General: To assess the virucidal efficacy of povidone iodine (PVP-I) on COVID-19 virus located in the nasopharynx

Specific:

i. To evaluate the efficacy of povidone iodine (PVP-I) to removeCOVID-19 virus located in the nasopharynx

ii. To assess the adverse events of PVP-I

**Trial design:**

This is a single-center, open-label randomized clinical trial with a 7-arm parallel-group design.

**Participants:**

The study will be conducted at Dhaka Medical College Hospital, Dhaka, Bangladesh.

**Inclusion criteria:**

All RT-PCR-confirmed COVID-19 cases aged between 15-90 years with symptoms for the past 4 days will be screened. Those who give informed consent, are willing to participate, and accept being randomized to any assigned group will also be considered for final inclusion.

**Exclusion criteria:**

Patients with known sensitivity to PVP-I aqueous antiseptic solution or any of its listed excipients or previously diagnosed thyroid disease or who had a history of chronic renal failure: stage ≥3 by estimated glomerular filtration rate (eGFR) Modification of Diet in Renal Disease (MDRD) or had acute renal failure (KDIGO ≥stage 2: creatinine ≥2 times from the baseline) or patients who required invasive or noninvasive ventilation or planned within the next 6 hours were considered for exclusion. Moreover, lactating or pregnant women will also be restricted to include here.

**Intervention and comparator:**

This RCT consist of seven arms:

Arm-1 (intervention group): will receive povidone iodine (PVP-I) nasal irrigation (NI) at a concentration of 0.4%

Arm-2 (intervention group): will receive PVP-I nasal irrigation at a concentration of 0.5%

Arm-3 (intervention group): will receive PVP-I nasal irrigation at a concentration of 0.6%.

Arm-4 (intervention group): will receive PVP-I nasal spray (NS) at a concentration of 0.5%.

Arm-5 (intervention group): will receive PVP-I nasal spray at a concentration of 0.6%.

Arm-6 (placebo comparator group): will receive distilled water through NI

Arm-7 (Placebo comparator group): will receive distilled water through NS

The intervention arms will be compared to the placebo comparator arms. Other supportive and routine care will be the same in both groups.

**Main outcomes:**

The primary outcome is the proportion of cases that remain COVID-19 positive following the intervention. It will be assessed from 1 minutes to 15 minutes after the intervention. Any occurrence of adverse effects following the intervention will be documented as a secondary outcome.

**Randomization:**

The assignment to the study (intervention) or control (comparator) group will be allocated in equal numbers through randomization using random number generation in Microsoft Excel by a statistician who is not involved in the trial. The allocation scheme will be made by an independent statistician using a sealed envelope. The participants will be allocated immediately after the eligibility assessment and consenting procedures.

**Blinding (masking):**

This is an open-label clinical trial, and no blinding or masking will be performed.

**Numbers to be randomized (sample size):**

A total of 189 confirmed cases of COVID-19 will be randomized into seven groups. In each arm, a total of 27 participants will be recruited.

**Trial Status:**

The current trial protocol is Version 1.5 from September 10, 2020. Recruitment began September 30, 2020 and is anticipated to be completed, including data analysis by February 28, 2021.

**Trial registration:**

The trial protocol has been registered in the ClinicalTrials.gov on September 16, 2020. NCT Identifier number: NCT04549376.

**Full protocol:**

The full protocol is attached as an additional file, accessible from the Trials website (Additional file 1). In the interest in expediting the dissemination of this material, the familiar formatting has been eliminated; this letter serves as a summary of the key elements of the full protocol.

**Supplementary Information:**

The online version contains supplementary material available at 10.1186/s13063-020-04963-2.

## Supplementary Information

**Additional file 1.**

## Data Availability

The corresponding author has access to the final trial information, and the data will be available on reasonable request (contact: dr.jahid61@gmail.com).

