# Supplementary material for: Virucidal effect of povidone iodine on COVID-19 in the nasopharynx: A structured summary of a study protocol for an open-label randomized clinical trial
Source: Trials. 2021 Jan 4;22:2. doi: 10.1186/s13063-020-04963-2 (PMC7780075; doi:10.1186/s13063-020-04963-2)
Supplement: Supplementary file 1 — Additional file 1. [file 13063_2020_4963_MOESM1_ESM.docx]

**STUDY PROTOCOL**

**“Virucidal Effect of Povidone Iodine on COVID-19 in Vivo: an open label randomized clinical trial"**

**NCT Identifier number: NCT04549376**

**ERC Memo no: ERC-DMC-ECC-2020-93**

**Date: 11. 09. 2020**

**(Version-1.5)**

**Title of the study**

**“Virucidal Effect of Povidone Iodine on COVID-19 in Vivo: an open label randomized clinical trial"**

|  | **Organization's Unique Protocol ID:** | **PiRCC-1131-2020** |
| --- | --- | --- |
|  | **Principal Investigator:** | **Dr. Mostafa Kamal Arefin, MBBS, MCPS, FCPS**  Otolaryngologist  Dhaka Medical College Hospital  Bangladesh  Email_ [arefin61dmc@gmail.com](mailto:arefin61dmc@gmail.com)  Cell: +8801671748866 |
|  | **Co-Investigator** | **Prof Sk Nurul Fattah Rumi,**  Professor & Head,  Dept. of Otorhiniolaryngology & Head Neck Surgery,  Dhaka Medical College Hospital.  Cell: 01711452381  Email: rumi17dr@gmail.com  **Prof. Sultana Sahana Banu**  Professor & Head,  Dept. of Virology  Dhaka Medical College  Cell:01911733070  Email: sultana_aus@yahoo.com  **Brig. General Dr. AKM Nasir Uddin**  Director, Dhaka Medical College Hospital  Cell: 01715016984  Email: [dmch@hospi.dghs.gov.bd](mailto:dmch@hospi.dghs.gov.bd)  **Dr Md Shahnoor Islam**  Director,  Pi Research Consultancy Center  +8801719224083  **Dr. Mohammad Jahid Hasan, MBBS, MPH,** Executive Director, Pi Research Consultancy Center**,** Cell: 01757818973  [dr.jahid61@gmail.com](mailto:dr.jahid61@gmail.com)**,** [**jahid@pircc.org**](mailto:jahid@pircc.org) |
|  | **Place of the study:** | Dhaka Medical College Hospital |
|  | **Duration of the study:** | 4 months |
|  | **Study period:** | September to December, 2020 |
|  | **Type of study:** | Randomized clinical trial |
|  | **Total cost:** | Self-funded |
|  | **Signature of the Principal Investigator:** | Signature |
|  | **Signature of Head of the Institute** | Signature |

**Part-A**

**Title: “Virucidal Effect of Povidone Iodine on COVID-19 in Vivo: an open label randomized clinical trial"**

**Short title:** **Virucidal Effect of Povidone Iodine on COVID-19 in Vivo**

**Acronym: VEP-COV**

**Background:** It is an established fact that coronavirus spreads through respiratory droplets. Colonization of the virus in the oropharynx and/or nasopharynx is considered to be a major factor for transmissibility of the virus through respiratory secretions. Preventing colonization of the virus by administrating povidone iodine in the nasal passage is therefore a rational thought that is supported by recent evidence of the in vitro virucidal action of povidone iodine in severe acute respiratory syndrome coronavirus 2 (SARS CoV-2). Therefore, this study was designed to assess the virucidal effect of povidone iodine on the COVID-19 virus in vivo.

**Patients and Methods:** This open-label randomized clinical trial will be conducted at the Department of Otorhinolaryngology and Head Neck Surgery in collaboration with the Department of Virology and Department of Medicine in Dhaka Medical College Hospital (DMCH). The study will be conducted from September 2020 to December 2020. A total of 189 confirmed cases of COVID-19 disease proven by reverse transcription polymerase chain reaction (RT-PCR) testing will be enrolled in this study. Written informed consent will be ensured before participation. In case of no literacy, fingerprint will be considered for written permission. Consent will be sought from the legal guardian in case of minor or underaged. Formal ethical clearance will be obtained from the Ethical Review Committee (ERC) of Dhaka Medical College (DMC). All of the participants will be divided into seven Arms: Arm 1 will receive povidone iodine (PVP-I) nasal irrigation at a concentration of 0.4%, Arm 2 and Arm 3 will receive 0.5% and 0.6%, Arm 4 will receive PVP-I nasal spray at a concentration of 0.5% and Arm 5 will receive 0.6%. Arm 6 (placebo comparator group) will receive nasal irrigation with distilled water (DW), and Arm 7 (placebo comparator group) will receive nasal spray with distilled water. The contact time will be minimum 30 seconds. After the individual application of PVP-I and distilled water in the respective participants, they will be tested again for RT-PCR for COVID-19 from nasopharyngeal samples. All patients will be subjected to a detailed history, physical examination and adverse events. Computer generated randomization sequence will be followed for randomization. Data will be recorded in a semi structured questionnaire and will be analyzed by ‘R-4.0.2’ data analysis software.

**Conclusion:** It is expected that the study will reveal the virucidal activity with optimal contact time and concentration of nasal preparations of PVP-I against COVID-19. The results may help in prevention strategies for the general population, health care workers and COVID-19-positive patients.

**Part-B**

**Title: “Virucidal Effect of Povidone Iodine on COVID-19 in Vivo: an open label randomized clinical trial"**

**Introduction**

Severe acute respiratory syndrome coronavirus 2 (SARS-CoV-2) has emerged as a new pathogen of the coronavirus family responsible for the coronavirus disease 2019 (COVID19) pandemic. The number of infections has grown exponentially over a short period of time (Pelletier *et al.*, 2020; Zhao *et al.*, 2020). Within seven months of its emergence in December 2019, the virus has spread rapidly and infected at least 12 million people worldwide, causing over half a million deaths from the disease (WHO, 2020). SARS‐CoV‐2 is an enveloped, positive-sense, single-stranded RNA virus and belongs to the same class of beta-coronaviruses as SARS-CoV and MERS-CoV, the viruses responsible for the Severe Acute Respiratory Syndrome (SARS) 2003 and Middle East Respiratory Syndrome (MERS) 2012 epidemics, respectively (Anderson *et al.*, 2020; Zhu *et al.*, 2020). Current evidence indicates that human-to-human transmission primarily occurs through respiratory droplets from coughs or sneezes and/or physical contact. As the virus can persist on contaminated surfaces for days, infection can occur indirectly by touch transfer of virus from these surfaces or hands to the mucosa of the mouth, nose or eyes (Anderson *et al.*, 2020; Chin *et al.*, 2020).

Viral loads are high in the nasal cavity, nasopharynx and oropharynx. Nasal goblet and ciliated cells within the respiratory epithelium have the highest expression of angiotensin converting enzyme 2 (ACE 2), the main receptor of COVID-19. Multiple reports have demonstrated that the nasal cavity, nasopharynx and oropharynx are important routes of transmission. Aerosol-generating procedures can enhance this transmission via transit through these areas of high viral content, releasing aerosols that can remain in the air for up to 3 hours. Transmission can occur in subclinical asymptomatic carriers, symptomatic infected carriers and convalescent seroconverted patients (Doremalen *et al.*, 2020; Pelletier *et al.*, 2020; Zou *et al.*, 2020). There is a growing need to develop processes to reduce virus transmission, as standard precautions, including the donning of masks and gloves, may not be sufficient. Early experience with COVID-19 outbreaks in hospital and healthcare settings has led frontline providers to suggest nasal and oral application of PVP-I as part of a transmission reduction plan (Mady *et al.*, 2020).

Povidone-iodine (polyvinylpyrrolidone iodine, PVP-I) is a water-soluble complex of povidone, a carrier molecule, and iodine that has powerful microbicidal activity. PVP-I formulations have been widely used for >60 years because of their broad-spectrum antimicrobial activity and established safety profile (Anderson *et al.*, 2020). It is available in various preparations for use as a disinfectant for skin, hand and mucosal surfaces, as well as for wound treatment and eye applications (Eggers *et al.*, 2018). The antimicrobial action of PVP-I occurs after free iodine dissociates from the polymer complex. Once in the free form, iodine rapidly penetrates microbes, disrupts proteins and oxidizes nucleic acid structures. This interaction ultimately results in microbial death (Kirk-Bayley *et al.*, 2020).

Interest in the use of PVP-I against coronaviruses was first reported in response to the SARS and MERS outbreaks in the past decade. Commercially available 10% PVP-I solutions have been tested against human coronaviruses HCoV 229E, HCV0OC43, SARS and MERS18, although these commercial solutions are unsuitable for use in the nasal and oral cavities at commercially available concentrations. Homology with the current COVID-19 pathogen suggests that PVP-I might be effective, but there are no reported studies that have concluded the efficacy against SARS-CoV-2 for any PVP-I solutions (Pelletier *et al.*, 2020). Therefore, the purpose of the randomized clinical trial is to investigate the virucidal activity of povidone iodine on COVID-19 located on the mucosal surface of the oral cavity, oropharynx and nasopharynx among COVID-19-positive patients seeking health care from Dhaka Medical College Hospital.

**Rationale of the study**

Coronavirus disease 2019 (COVID-19) is a highly infectious disease that causes human-to-human transmission primarily by respiratory droplets from coughing, sneezing and speaking. In early SARS-CoV-2 infection, a greater viral titre can be found in saliva and nasal mucous; minimization of these titres should help to reduce cross infection. Povidone-iodine (PVP-I) disinfectant has better antiviral activity than other antiseptics and has already been proven to be an extremely effective virucide in vitro against severe acute respiratory syndrome and Middle East respiratory syndrome coronaviruses (SARS-CoV and MERS-CoV). Its in vivo virucidal activity is unknown, although its nasal or oral preparation can be effective in reducing transmission. Earlier, very few studies evaluated the efficacy of povidone-iodine as a nasal antiseptic or oral rinse antiseptic against the SARS-CoV-2 virus, but the results of those studies are not undebatable. Therefore, the aim of this study was to investigate the virucidal activity of povidone iodine on coronaviruses located on the mucosal surface of the oropharynx and nasopharynx, which is very rational in this pandemic period.

**Research Hypothesis:**

**Null hypothesis:** Administration of nasal preparations of povidone-iodine (PVP-I) is no longer effective in clearing COVID-19 virus located on the mucosal surface of the nasopharynx.

**Alternative Hypothesis:** Nasal preparations of povidone-iodine (PVP-I) can effectively clear COVID-19 virus located on the mucosal surface of the nasopharynx.

**Objectives:**

**General Objective:**

To determine the virucidal efficacy of povidone iodine on COVID-19 virus located in nasopharynx.

**Secondary objectives:**

1. To determine the efficacy of povidone iodine in clearing COVID-19 virus located in the nasopharynx
2. To assess the adverse events among the groups

**METHODOLOGY**

**Study Design:** Open label randomized clinical trial.

**Place of Study:** Department of Otorhinolaryngology and Head Neck Surgery, in collaboration with Department of Virology and Department of Medicine, Dhaka Medical College Hospital, Dhaka, Bangladesh.

**Period of study:** The study period will be September 2020 to December 2020.

**Study population:** Confirmed cases of COVID-19 disease proven by RT-PCR testing.

**Sampling and statistical basis of the sample size:** The sample size was determined using the following formula:

**Sample size:**

$$n=(\frac{r+1}{r})\frac{\sigma^{2}(Z_{\beta}+\text{Z}_{\alpha\text{/2}})^{2}}{\text{(}d\text{ifference})^{2}}$$

- - For 80% power, Z_β_=.84
  - For 0.05 significance level, Z_α_=1.96
  - r=1 (equal number of cases and control)
  - σ=10.0
  - Difference = 5.0

Therefore, n=126

Including exclusion criteria total sample 63*2+

30*2=186.

Therefore, in each group, a total of 27 samples will be collected after randomization.

**Number of participants:** The total number of participants will be 27 in each arm (7 Arm x 27=a total of 189 sample).

**Inclusion criteria:**

- - - - Age: 15-90 years
      - Either gender
      - Patients diagnosed with COVID-19 disease by RT-PCR
      - Have confirmed COVID-19 symptoms and symptom onset within the past 4 days
      - Capable of using a nasal spray device and perform nasal irrigation required by the study
      - Willing to participate

**Exclusion criteria:**

- Patients with known sensitivity to PVP-I aqueous antiseptic solution or any of its listed excipients
- Previously diagnosed thyroid disease
- Patients with chronic renal failure: stage ≥3 by estimated glomerular filtration rate (eGFR) Modification of Diet in Renal Disease (MDRD)
- Patients with acute renal failure (KDIGO ≥stage 2: creatinine ≥2 X baseline)
- Pregnant and lactating mother
- Current requirement for invasive or noninvasive ventilation or planned within the next 6 hours.

**Operational definitions:**

**Povidone Iodine (Kirk-Bayley *et al.*, 2020):**

Povidone-iodine (iodine with the water-soluble polymer polyvinylpyrrolidone, PVP-I) was discovered in 1955 at the Industrial Toxicology Laboratories in Philadelphia by H. A. Shelanski and M. V. Shelanski. It was developed in order to find an antimicrobial iodine complex that was less toxic than tincture of iodine, which caused burn. PVP-I antibacterial activity is enhanced by dilution of the usually available 10% w/w cutaneous solution, from 1:2 dilution up to a 1:100 dilution (0·1%).

**Method and data collection:**

**Participant selection and enrolment:** All patients admitted to the medical wards through the emergency department or outpatient department with suspected COVID-19 cases will be initially approached and screened for confirmation of COVID-19. Confirmation of COVID-19 will be performed by positive impression of RT-PCR results. Following confirmation of the viral infection, the patients will be seen by the ward doctors and clinical trial physicians (registered physicians). Moreover, baseline complete blood count (CBC) will be performed for each patient. Chest X-ray/high-resolution computed tomography (HRCT) will be performed in necessary cases. The ward doctors and trial physicians will together care for the patient; then, the patients will be briefed about the aim, objective and details of the procedure of the study. If without capacity, their relatives will be approached about the trial and seek their consent for recruitment. As this study will be confined to a tertiary care hospital, patients will be recruited up to day 4 of illness.

**Consenting participants:** It will be prioritized that the patients will be treated early for COVID-19 management by a standard regimen and in accordance with the guidance of the national guidelines. Within the shortest possible time, preferably within 4 hours of admission, the consenting procedure will be completed either from the patient or from the attendant/legal guardian of the patients to obtain the full potential benefit of the treatment. Written informed consent will be obtained from the patient in their own language [Bengali] by trial physicians working on the trial.

**Screening for eligibility:** During admission, demographic and clinical details, including name, age/date of birth, sex, duration of onset of fever and hospitalization, vital statistics will be recorded before randomization. RT-PCR for COVID-19 will be used for confirmation of COVID-19 virus infection. A screening log will be maintained including these essential details and the decision made by the patient, attendant or legal guardian where appropriate concerning recruitment. Ineligible and non-recruited patients will also be cared for by ward physicians following standard treatment guidelines.

**Procedure of randomization:** The study participants will be randomly assigned in this study into seven (7) equal Arm, where each arm has equal opportunity to receive any treatment option. Randomization will be performed by computer generated random sequences.

- - Arm 1 will receive povidone iodine (PVP-I) nasal irrigation at a concentration of 0.4%
  - Arm 2 will receive povidone iodine (PVP-I) nasal irrigation at a concentration of 0.5%
  - Arm 3 will receive povidone iodine (PVP-I) nasal irrigation at a concentration of 0.6%
  - Arm 4 will receive PVP-I nasal spray at a concentration of 0.5% with standard care.
  - Arm 5 will receive PVP-I nasal spray at a concentration of 0.6%
  - Arm 6 (placebo comparator group) will receive nasal irrigation with distilled water
  - Arm 7 (placebo comparator group) will receive nasal spray with distilled water

This can be done quickly, with baseline information typically captured in <15 min. Recruitment & randomization will be performed contemporaneously with assessment/resuscitation of patients by a second person so that medical care is not delayed by recruitment. Patients will only be randomized once baseline data have been entered; after this has been done, participants will not be withdrawn from the study. Following tossing, the trial physicians reported the principal investigator (PI) of the trial, and then the PI handed over an opaque sealed envelope, which will be numbered sequentially to correspond to patient enrolment numbers, which will be used for concealment of treatment allocations. Envelopes will be kept in a locked drawer and will be opened in strict numerical order by supervisor in the presence of the nursing supervisor of the study unit. Allocation between arms will initially be equal, although this may be revised following guidance of the PI and his team (as per the statistical plan).

**Withdrawal of study participants:** Participants are free to withdraw from the study at any point, for any reason. If this occurs, the primary reason for withdrawal will be documented in the participant’s case record form. The participant will have the option of withdrawal from:

- Study medication with continued study procedures and collection of clinical and safety data,
- All aspects of the trial but continued use of data collected up to that point,
- All aspects of the trial with the removal of all previously collected data, or
- All aspects of the trial with the removal of previously collected data and stored participant samples.

Randomized patients who wish to withdraw from the study before they have undertaken any study-related procedures will be replaced. Data on the original participant will be kept on the CRF/database if the participant agrees to this.

**Investigational medicinal product and placebo:**

**Intervention for Arms 1, 2, 3, 4 and 5 (PVP-I group/treatment group):** Arm 1 will receive povidone iodine (PVP-I) nasal irrigation at a concentration of 0.4%, Arm 2 will receive povidone iodine (PVP-I) nasal irrigation at a concentration of 0.5%, Arm 3 will receive povidone iodine (PVP-I) nasal irrigation at a concentration of 0.6%, Arm 4 will receive PVP-I nasal spray at a concentration of 0.5% and Arm 5 will receive PVP-I nasal spray at a concentration of 0.6%. The contact time will be minimum 30 seconds.

**Intervention for Arms 6 and 7 (placebo comparator):** Arm 6 (Placebo comparator group) will receive nasal irrigation with distilled water, and Arm 7 (Placebo comparator group) will receive nasal spray with distilled water for the same duration as a placebo agent.

All groups of patients will receive standard medical care according to country guideline.

**Preparation of placebo:** As this study will be an open label study, no blinding will be performed and no dummy placebo will be made.

### Storage:

Drugs will be stored at room temperature 20-25^0^ in a trial storage room. The reconstituted solution will also be kept at temperatures of 20-25^0^. Solution should be used within 48 hours after mixing. The trial storage room will be monitored, and the logging of temperatures will be noted. Drugs will be transported by the manufacturers to the study sites using their standard temperature-controlled processes.

**Dosing regimen:**

**Intervention Arms (Arms 1, 2, 3, 4, 5):** Participants allocated to PVP-I solution (especially prepared for the study) after confirmation of COVID-19 positivity. Proper dilution with normal saline will be added, which will be physically and chemically stable for 6 hours. Other supporting care (crystalloid solution infusion), and monitoring of vital statistics, in case of bleeding protective measures will be performed according to the national guidelines.

**Control Arms (Arms 6 and 7):** Participants allocated distilled water for nasal irrigation for Arm 6 a placebo that will also be reconstituted and applied at the same rate as mentioned in the invention groups and distilled water for nasal spray for Arm 7 a placebo that will also be reconstituted and applied at the same rate as mentioned in the invention groups.

In both Arms of patients, 10 ml of PVP-I or distilled water will be administered for 2 minutes. Nasal spray will be administered in each nostril using an atomizing device (2 sprays for average device). Control groups, will receive distilled water irrigation and distilled water nasal spray at similar amounts and durations. Further RT-PCR will be assessed within 1 minutes to 15 minutes of the following intervention.

**Participant compliance:** The study team will not create any problem with compliance while the patient is under care and supervision in the hospital. It will be ensured that all recruited patients will receive the appropriate interventions, although they could be interrupted due to the onset of potential adverse effects such as hypotension.

**Overdose:** The study team will titrate the drugs against the key adverse effect of hypotension. Nasal spray and nasal irrigation will be halted if patients complain of significant discomfort or clinically significant hypotension or signs of anaphylaxis. Procedures will be started again at a lower rate if the condition settles or according to the patient’s choice.

**Responsibility sharing of care of the patients:** As the patients will remain under the care of the hospitals’ consultant physicians/unit head who will have primary responsibility for their management. Management protocols will be agreed upon between the medical team and study team. Decisions about intubation, requirement of blood and blood products, transfer of patients to intensive care, weaning of ventilation and extubation will be made by the medical team independently of study doctors. All decisions will be based on the patient’s clinical condition and the available hospital resources, as per usual hospital practice. The study team preferably by trial physicians will note down the record in the patient’s case record form (CRF).

**Prohibited Medications:** No medicines are specifically prohibited for this clinical trial.

**Non-investigational medicinal products:** All patients will receive standard care plus supporting care according to the “National Guidelines on Clinical Management of Coronavirus Disease 2019 (COVID-19)”. Among the standard care fluids, oxygen, ventilatory support, and antipyretics will be provided as necessary in all Arms of patients and will be decided by the chief/consultant of the admission unit. No other noninvestigational product will be used for the study purpose. In case of any product required for the best suited management, inclusion of this patient will be decided upon the opinion of the PI.

**Methods of data collection:** Data collection will be performed by the trial physicians. A paper based semi-structured questionnaire will be made and pretested before data collection. All potential participants will be recorded in this database at first contact and assigned a study number. This study number (e.g., DMC-PVP-I-0001) will be used for all data and samples collected from that person. Study doctors will recruit and randomize patients. They will not be able to predict allocation before randomization. Following randomization, allocation will be performed by the PI, and trial physicians or nurses will confirm the administration of either drug (0.4% or 0.5% or 0.6% PVP-I)/placebo (distilled water). Patients will be closely monitored. Temperature will be measured every 6 hours, and blood pressure, respiratory rate, and pulse rate will be measured every hour. Urine output will be measured in every 8 hours. Drug-related side effects or any adverse events will be observed in each group.

If there is no reaction or unusual event, another RT-PCR sample will be collected from the nasopharynx and sent for further testing.

The total duration of the follow-up will be 2 days (or a person fulfills the discharge criteria). All clinical events will be noted in case record form by trial physicians, including vital status at hospital discharge. Data entry will be date/time stamped. Laboratory analysis data will be collected using the same CRF.

**Data quality and standards:** Following collection, all data sets and collected record forms will be checked. A formal clinical data management plan will be written before the study starts, agreed upon by the co-investigators and Data Monitoring Committee, and documented as a trial master file. Laboratory results will be discussed regularly between the PI and laboratory managers.

**Proposed Analysis Plan of the study:**

Data analysis will be performed by data management software, R-4.0.2’. Then, the principal investigator (PI) will also check and verify the data. Then data will be transferred to statistician. If it is consistent with the objectives, then the PI and his team will draft the final result.

The following procedure will be followed during data analysis. The RCT aims to determine whether povidone iodine can clear COVID-19 virus from the nasopharynx of COVID-19 positive patients. The analysis will be performed on an intention-to-treat basis and a per-protocol basis. Subgroup analyses based on the primary outcome will also be estimated. Although the study is not formally powered for these subgroup analyses, the analysis will be added to draw a more conclusive statement. Statistical significance is set at the 95% confidence level at the 5% acceptable error level. Differences will be considered significant at the P < 0.05 level for all these tests. The data will be expressed as the means ± standard deviations (SD) for continuous variables and as frequencies (%) for categorical variables. Chi-square test and Student’s t test or ANOVA will be used in cases of normally distributed data, and similar non parametric data will be used whenever appropriate. Data will be analyzed by SPSS 20 Windows version (Chicago, Illinois, USA), and graphs and charts will be expressed by Microsoft Excel 2016.

.

**Ethical consideration:**

The researcher is duly concerned about the ethical issues related to the study. In this study, the following criteria will be followed to ensure maintaining ethical values. All ethical measures will be followed in accordance with the current Declaration of Helsinki and Good Clinical Practice (GCP) guidelines.

1. Formal ethical clearance was taken from the ethical review committee of the Dhaka Medical College for conducting the study.
2. Confidentiality of the person and the information will be maintained, observed and unauthorized persons will have no access to the data.
3. Informed written consent will be obtained from the subject.
4. The content of the consent requirements will be as such:
   - 1. Explanation of the nature & purpose of the study.
     2. Explanation of the procedure of study.
     3. Explanation that they have the right to refuse, accept & withdraw to participate in the study.
5. It will be clear to the patient that his/her confidentiality will be maintained, that there is no monetary benefit of the patient in this study, he/she can refuse to participate in the study at any time and it will in no way hamper his/her treatment.
6. It will be made clear to the patient that there is an invasive procedure that will be performed for the study, mostly for the sample collection of RT-PCR.
7. They will be assured that any adverse reaction or unusual events will be managed immediately, with highest care.

**Reference:**

Anderson, D. E. *et al.* (2020) ‘Povidone-Iodine Demonstrates Rapid In Vitro Virucidal Activity Against SARS-CoV-2, The Virus Causing COVID-19 Disease.’, *Infectious diseases and therapy*, pp. 1–8.

Chin, A. W. H. *et al.* (2020) ‘Stability of SARS-CoV-2 in different environmental conditions’, *The Lancet Microbe*. Elsevier Ltd, 1(1), p. e10.

Doremalen, N. van *et al.* (2020) ‘Aerosol and Surface Stability of SARS-CoV-2 as Compared with SARS-CoV-1’, *The New England Journal of Medicine*, 382(16), pp. 1–3.

Eggers, M. *et al.* (2018) ‘In Vitro Bactericidal and Virucidal Efficacy of Povidone-Iodine Gargle/Mouthwash Against Respiratory and Oral Tract Pathogens’, *Infectious Diseases and Therapy*. Springer Healthcare, 7(2), pp. 249–259.

Kirk-Bayley, J. *et al.* (2020) ‘The Use of Povidone Iodine Nasal Spray and Mouthwash During the Current COVID-19 Pandemic May Protect Healthcare Workers and Reduce Cross Infection.’, *SSRN Electronic Journal*, pp. 1–10.

Mady, L. J. *et al.* (2020) ‘Consideration of povidone-iodine as a public health intervention for COVID-19: Utilization as “Personal Protective Equipment” for frontline providers exposed in high-risk head and neck and skull base oncology care’, *Oral Oncology*, p. 104724.

Pelletier, J. *et al.* (2020) ‘Efficacy of Povidone-Iodine Nasal And Oral Antiseptic Preparations Against Severe Acute Respiratory Syndrome-Coronavirus 2 (SARS-CoV-2)’, *Medrxiv*, 2, pp. 1–7.

WHO (2020) *WHO Coronavirus Disease (COVID-19) Dashboard*.

Zhao, S. *et al.* (2020) ‘Preliminary estimation of the basic reproduction number of novel coronavirus (2019-nCoV) in China, from 2019 to 2020: A data-driven analysis in the early phase of the outbreak’, *International Journal of Infectious Diseases*, 92(January), pp. 214–217.

Zhu, N. *et al.* (2020) ‘A novel coronavirus from patients with pneumonia in China, 2019’, *New England Journal of Medicine*, 382(8), pp. 727–733.

Zou, L. *et al.* (2020) ‘SARS-CoV-2 Viral Load in Upper Respiratory Specimens of Infected Patients’, *New England Journal of Medicine*, 382(12), pp. 1175–1177.

**BUDGET:** Self-funded.

**Time line:**

| **Task** | **September** | **October** | **November** | **December** |
| --- | --- | --- | --- | --- |
| **Recruitments** |  |  |  |  |
| **Data analysis** |  |  |  |  |
| **Manuscript draft** |  |  |  |  |
| **Final draft and dissemination** |  |  |  |  |
